# Bioimpedance spinal needle provides high success and low complication rate in lumbar punctures of pediatric patients with acute lymphoblastic leukemia

**DOI:** 10.1038/s41598-022-10915-4

**Published:** 2022-04-26

**Authors:** Satu Långström, Anu Huurre, Juho Kari, Olli Lohi, Harri Sievänen, Sauli Palmu

**Affiliations:** 1 Department of Pediatric Hematology, Oncology and Stem Cell Transplantation, Helsinki University Hospital, New Children’s Hospital, Helsinki, Finland; 2 Department of Pediatric Hematology and Oncology, Turku University Hospital, Turku, Finland; 3 Injeq Oy, Tampere, Finland; 4 Tampere Center for Child, Adolescent and Maternal Health Research, Faculty of Medicine and Health Technology, Tampere University, and Cancer Center, Tampere University Hospital, Tampere, Finland

**Keywords:** Biophysics, Health care, Oncology

## Abstract

In this prospective single-arm study of 50 pediatric patients with acute lymphoblastic leukemia (ALL), we evaluated the clinical performance of a novel bioimpedance spinal needle system in 152 intrathecal treatment lumbar punctures (LP) of these patients. The system detects in real-time when the needle tip reaches the cerebrospinal fluid (CSF) in the spinal canal. The success was defined as getting a CSF sample and/or administering the intrathecal treatment with one needle insertion. Incidence of traumatic LP (TLP) was defined as ≥ 10 erythrocytes/µL of CSF. Post-procedural complications were monitored with a one-week diary and one-month register follow-up. The success of the first attempt was 79.5%, with the CSF detection sensitivity of 86.1%. The incidence of TLP was 17.3%. A successful first attempt was associated with a significantly lower incidence of TLP (10% vs 40%, p = 0.0015). During the week after the procedure, the incidence of post-dural puncture headache was 6%. During the follow-up, no major complications were observed. In conclusion, the novel bioimpedance spinal needle system achieved a high success rate and low incidence of TLP and other complications in pediatric patients with ALL in a real-world clinical setting, indicating clinical utility for this system in pediatric hemato-oncology.

## Introduction

Lumbar puncture (LP) is an essential clinical procedure for diagnostics of acute lymphoblastic leukemia (ALL), and intrathecal administration of chemotherapy in patients with ALL. Traumatic LP (TLP), manifesting as red blood cells in the sample of cerebrospinal fluid (CSF) above a specified criterion, is common in clinical practice. Compiling the reported data from almost 19,000 LP procedures performed in pediatric oncology patients^1–9^, the mean occurrence of TLP, defined as ≥ 10 erythrocytes/µL of CSF, was 19% varying from 1^6^ to 36%^4,7^. These reported values mostly reflect the incidence of TLPs in the patient’s first diagnostic puncture, not in intrathecal treatment punctures during the subsequent therapy period. Since blood leakage into the spinal canal not only compromises the diagnostics but also increases the risk of blasts entering CSF space and the central nervous system, a successful puncture at the first attempt together with a low incidence of TLP are of paramount importance. TLP and a high number of blasts in CSF are associated with patients’ poorer event-free survival and relapse of the disease^1,3–5,7,9^.

Albeit LP is a well-established and safe procedure some complications are relatively common and inconvenient to the patient. The most considerable LP-specific complication is post-dural puncture headache (PDPH)^10^. In children with ALL, the incidence of PDPH is yet quite low varying from 5 to 14%^11–15^. Excellent success at the first attempt is likely to contribute to the low incidence of PDPH^14^.

Success at the first attempt cannot be taken for granted, however. In pediatric patients with ALL, the success of LP with single skin penetration is generally good varying from 70^13^ to 90% or more^14,15^. In adults with leukemia, the success is associated with about halved incidence of TLP^16^, but to our knowledge, this issue is not specifically addressed in children with ALL. Generally, in pediatric patients, a successful LP at the first attempt is associated with about 50% lower incidence of TLP as well^17^.

The success of LP can be improved by employing image guidance either before inserting the needle into the body or in real-time during the procedure. Crucial to the utility of image guidance is whether the obtained information on the patient’s anatomy or the orientation and location of the advancing needle can help prevent puncture-induced trauma on tissues in contact with CSF. According to recent meta-analyses^18–20^, ultrasound guidance confers several benefits: it can at least halve the incidence of TLPs, improve the success rate of LPs, and reduce the incidence of failed procedures compared to traditional palpation-based LP. As regards the children with ALL, challenging patients with high body mass index (BMI), anatomic abnormalities, young age, or a history of several failed attempts may be referred to ultrasound imaging for guidance before or during the LP procedure^18,21^.

While there is convincing evidence for the clinical utility of ultrasound imaging in performing LP^18–20^, particularly the real-time guidance requires the physician’s one hand to use the ultrasonic probe and the other to advance the needle, let alone the need for bedside imaging equipment, and user’s adequate proficiency to use this technique. These needs may alter the performance of the proven LP the physicians are commonly used to doing. Thus, a needle guidance method that does not require specific expertise and simultaneous operation of the imaging equipment but provides real-time feedback on the needle tip location might be an option worth considering. Recently, a novel bioimpedance-based spinal needle system was found clinically feasible in LPs of both adults and infants, including neonates^22,23^. This new system provides real-time needle guidance by informing the user when the needle tip reaches CSF in the subarachnoid space of the spinal canal, and the correct location for CSF sampling or administration of intrathecal treatment. In this prospective study, we extended the use of the bioimpedance spinal needle system to LPs of pediatric patients with ALL and specifically evaluated the success rate at the first attempt, the incidence of TLP and other potential complications related to its use in a real-world clinical setting.

## Methods

### Participants

All pediatric patients aged between 18 months and 18 years treated for ALL in pediatric hemato-oncology departments of Helsinki, Turku, and Tampere university hospitals were eligible for the study. LP procedures and data collection were done between November 2019 and September 2020. Before the study, all patients and/or their parents gave informed consent, depending on the patient’s age.

Exclusion criteria were refusal to participate, no informed consent from the patient and/or parents as appropriate for age, or any concurrent clinical contraindication including skin infection around the puncture site, unstable hemodynamics, bleeding tendency, or increased intracranial pressure. Also, the patient’s first diagnostic LP was excluded because of apparent ethical and practical reasons. According to the study protocol, our goal was to gather data from at least 150 procedures of at least 50 pediatric patients with ALL but not more than four procedures per patient.

### Bioimpedance spinal needle system

The bioimpedance spinal needle system (Injeq IQ-Tip system, Injeq Oy, Tampere, Finland, www.injeq.com) comprises either 40 mm, 65 mm, or 90 mm long 22G Quincke-type spinal needles (IQ-Tip spinal needle), bioimpedance analyzer, and a thin, flexible coaxial cable for connecting the needle to the analyzer. The needle is practically identical to the Quincke-type spinal needle with a stylet, except the stylet is configured as a bioimpedance electrode and has a cable connector at the handle end. Basically, performing the LP either with the bioimpedance needle or conventional spinal needle is similar. Also, the removal and reinsertion of the stylet do not differ from the conventional spinal needle.

For the needle guidance, the bioimpedance spinal needle system measures continuously the bioimpedance of tissues that are in immediate contact with the needle tip. In short, the analyzer employs a 1 ms long proprietary pulse sequence as an excitation signal which is continuously transmitted to the needle electrode. This low-power signal carries most of its power at few specified frequencies within a range from 1 to 349 kHz. Simultaneously the bioimpedance of a small tissue volume (< 1 mm^3^) around the needle tip is calculated by discrete Fourier-transformations of the measured voltage and current data at the specified frequencies^24^. These bioimpedance values are used for tissue classification 200 times per second, meaning that the tissue classification is performed virtually in real-time (at every 5th ms) with high spatial sensitivity (< 1 mm^3^). By design, the system gives an audio-visual alarm when the needle tip reaches CSF in the subarachnoid space.

In addition, the raw bioimpedance data were stored into the analyzer memory for further post-hoc analysis.

### Clinical procedures

Before starting the study, the manufacturer’s representatives (JK and HS) gave an overview and practical training session on the use of the bioimpedance spinal needle system to local principal investigators (SL, AH, and SP), other physicians, and research nurses. The attendees could try the system on a lumbar phantom (Blue Phantom BPLP2201, CAE Healthcare, FL, USA). Later, the principal investigators were responsible for the training of new users at their departments.

All study LP procedures were part of the patients’ scheduled intrathecal treatment protocol. The procedure was performed under general anesthesia in the lateral decubitus position. The personnel of the participating departments performed the LP procedures according to their normal treatment schedules and practices. Primarily, all study procedures were to be performed with the bioimpedance needle system. However, in case of two unsuccessful attempts with the system, the user (i.e., the physician who performed the study LP) could, upon personal decision, use the conventional Quincke-type 22G spinal needle to complete the procedure.

After perceiving the CSF detection alarm from the system and upon clinical judgment, the user could either remove the stylet and check for CSF flow through the needle or continue the puncture. Also, the user could remove the stylet and check for CSF flow solely upon the clinical judgment about the location of the needle tip before the alarm.

When the needle tip was correctly in the subarachnoid space verified by CSF flow, the user took the CSF sample and delivered the intrathecal treatment according to the patient’s treatment protocol.

The red blood cell count (erythrocytes/µL) was routinely determined from the second or the third vial of CSF sample by the hospital laboratories with standard cytometric methods.

### System performance

Immediately after the study LP, the user evaluated the system performance and recorded the data on the patient’s Case Report Form (CRF) concerning the success of LP at the first attempt, the total number of attempts needed for a successful procedure, and the accuracy of perceived alarms.

The success of LP at the first attempt was defined as getting a valid CSF sample and/or delivering intrathecal treatment with single needle insertion and skin penetration. Redirections of the needle inside the body, if needed, were allowed.

The accuracy of CSF detection was evaluated by counting the numbers of correct alarms (i.e., there was an alarm and CSF flew after the stylet removal), missing alarms (i.e., there was no alarm, but CSF flew after the stylet removal), and false alarms (i.e., there was an alarm, but CSF did not flow after the stylet removal).

The CSF detection sensitivity and false detection rate were determined as performance outcomes. The CSF detection sensitivity was calculated by dividing the number of correct alarms by the sum of correct alarms and missing alarms. The false detection rate was calculated by dividing the number of procedures showing false alarms by the number of all procedures.

### Post-procedural complications and symptoms

After each study LP procedure, the research nurse gave the patient/parents a diary to be filled for reporting perceived symptoms or complications during the day of the procedure and seven subsequent days. These included PDPH, headache, nausea, backache, fever, leaking or inflammation of the puncture site. PDPH was defined as a severe headache that worsened in sitting or standing position, eased after lying down, and occurred within 7 days of the preceding LP. After the week, the research nurse called the patient/parents and inquired about the diary data.

Concerning the four weeks after the study LP procedure, the research nurse or investigator searched the patient-specific hospital records for potential complications or issues that might have been related to the LP procedure or the use of the bioimpedance needle system. Potential causality between the found complications or symptoms and the LP procedure or the use of the bioimpedance spinal needle system was evaluated by the local principal investigator using 5-level scoring system (not related, unlikely, possible, probable, and causal).

### Statistical analysis

Mean, standard deviation (SD) and ranges are given as descriptive data. A cumulative distribution curve was specifically used to illustrate the incidence of TLP. The curve depicts respective proportions of CSF samples, where the erythrocyte counts did or did not indicate TLP at any given criterion. In this study, the standard criterion of ≥ 10 erythrocytes/µL of CSF used in hemato-oncology was chosen as the criterion for TLP^2^.

As a primary analysis, the association of the success of LP at the first attempt with the incidence of TLP was evaluated. Then, associations of the physician’s experience and patients’ body weight with the success rate and incidence of TLP were assessed. The patient’s weight was categorized into underweight (BMI < 17 kg/m^2^), normal weight (17 ≤ BMI < 25 kg/m^2^), overweight (25 ≤ BMI < 30 kg/m^2^), and obese (BMI ≥ 30 kg/m^2^) by applying the patients’ BMI adjusted for age^25^. Finally, the association of system performance with the incidence of TLP was assessed.

The statistical comparisons were done using the Χ^2^-test, and a p-value less than 0.05 was considered statistically significant. The 95% confidence intervals (95% CI) of the success rate at the first attempt, CSF detection sensitivity, and false detection rate were calculated with the exact Clopper-Pearson method. Statistical analyses were done with IBM SPSS Statistics for Windows, version 27.0 (IBM Corp., Armonk, NY, USA).

### Ethics and regulatory issues

The study protocol was approved by the Regional Ethics Committee of the Expert Responsibility Area of Tampere University Hospital, Finland (R19050). Also, the National Competent Authority (Valvira, Helsinki) was notified about the study before its commencement in accordance with national legislation. The study was registered in ClinicalTrials.gov (date 28/08/2019, NCT04070144) and conducted according to Helsinki Declaration and Good Clinical Practice.

## Results

### Patient characteristics

The study LP procedures were performed in different phases of patients’ intrathecal treatment schedules, excluding the patients’ first diagnostic punctures. In total, data from 152 procedures of 50 pediatric patients with ALL (27 males and 23 females) were obtained. The present data comprised four LP procedures from 26 patients, three from 8 patients, two from 8 patients, and one from 8 patients. A valid CSF sample was obtained in 150 procedures and intrathecal treatment was delivered in 151 procedures.

Patients’ age ranged from 1.8 to 16.0 years with a mean of 7.1 (SD 3.9) years. Their body height ranged from 85 to 178 cm with a mean of 123 (24.3) cm, and weight from 13 to 71 kg with a mean of 29 (16.0) kg. At the time of their study LP procedures, 4% of patients were underweight, 66% normal weight, 23% overweight, and 7% obese.

### Success of LP and incidence of TLP

Of the 151 LP procedures, 120 were successful at the first attempt and the respective success rate was 79.5% (95% CI from 72.1 to 85.6%). One procedure was excluded from this analysis, because the physician accidentally chose too short a needle for the first attempt. Two attempts were required in 20 procedures, three attempts in five procedures, and at least four attempts in six procedures. One procedure could not be completed even after several attempts by three different physicians.

The incidence of TLP was 17.3% (95% CI 11.7–24.4%). The red blood cell count in the CSF sample was at least 10 erythrocytes/µL in 26 samples out of 150 samples. The incidence of erythrocyte-free samples was 55.3%.

**Figure 1:**
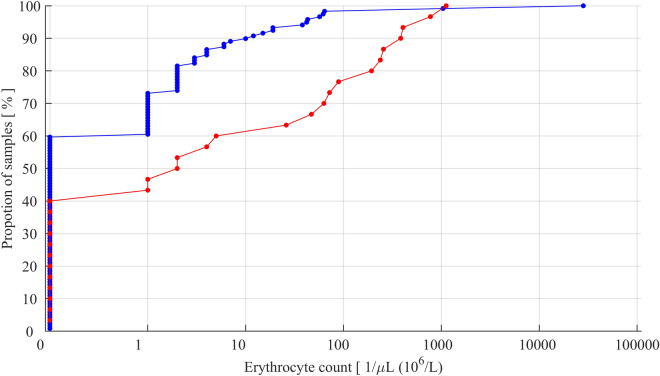
Cumulative distribution curves of erythrocyte count data obtained from the study LP procedures that were successful at the first attempt (blue line) and not successful at the first attempt (red line).

The success rate at the first attempt was associated with a significantly lower incidence of TLP (Fig. 1). When the first attempt was successful, the incidence of TLP was 10.9% in contrast to 40.0% when more attempts were needed (Χ^2^ test, p = 0.0015).

The success rate at the first attempt was similar when the normal and underweight patients were compared to overweight and obese patients (79.2% vs. 80.0%, Χ^2^ test, p = 0.91). The respective incidences of TLP were 18.7% and 13.3% (Χ^2^ test, p = 0.42).

Twenty-six different physicians performed at least one study LP procedure. The median number of procedures per physician was 4, ranging from one to 16. Physicians with previous experience of more than 100 LPs performed 45% of the study procedures. The success rate at the first attempt did not differ between the more experienced physicians and less experienced physicians (76.1% vs 82.1%, Χ^2^ test, p = 0.37). The incidence of TLP was 13.2% in LPs performed by the experienced physicians and 20.7% in LPs performed by less experienced physicians (Χ^2^ test, p = 0.23).

### System performance and incidence of TLP

Subarachnoid space was verifiably reached with the bioimpedance needle system in 137 procedures out of 151 completed procedures (91%). The system detected CSF correctly in 118 out of these 137 procedures (86.1%, 95% CI 79.2–91.4), whereas there was no CSF alarm in 19 procedures.

False CSF alarms occurred in 14 out of 152 procedures (9.2%, 95% CI 5.1–15.0). In six out of these 14 procedures, the system detected CSF correctly after preceding false alarms. Seven procedures were eventually completed with a conventional spinal needle.

**Figure 2:**
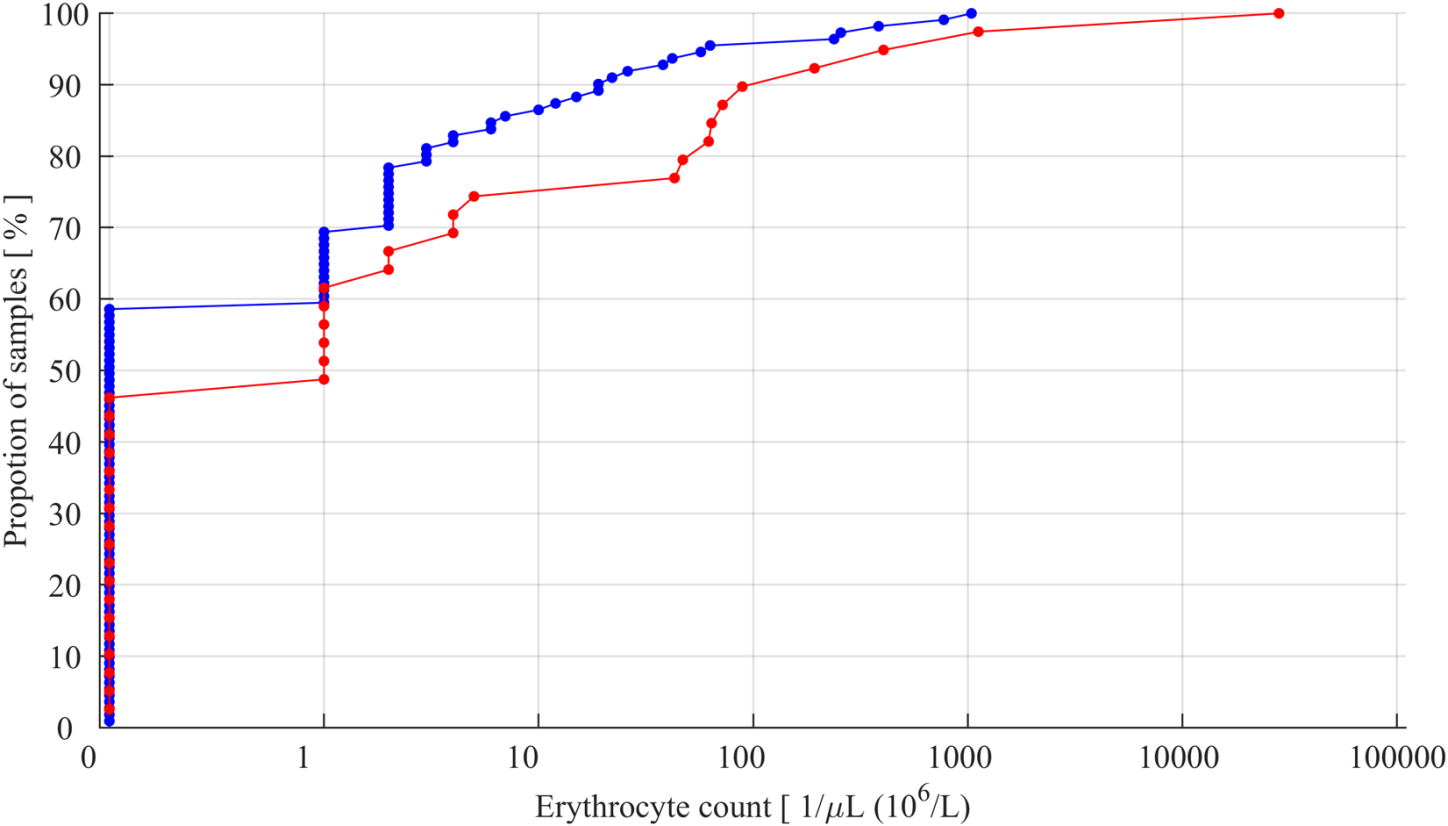
Cumulative distribution curves of erythrocyte count data obtained from the study LP procedures when the performance of the bioimpedance needle system was correct (blue line) or there was false or missed detections (red line).

Correct performance of the system seemed to be associated with a lower incidence of TLP (Fig. 2). The incidence of TLP was 14.4% when CSF was correctly detected in contrast to the 25.6% incidence when there were false or missing CSF alarms (Χ^2^ test, p = 0.11).

### Post-procedural complications

The 7-day diary data were received after 140 study LP procedures (92%). During the 7-day period, PDPH was observed after 9 procedures (6%), three times in the same patient. Other complications during the first week included nausea after 22 procedures (15%), headache after 20 procedures (14%), backache after 20 procedures (14%), and fever after 10 procedures (7%).

Patients’ four-week register data were accessed for all 152 procedures (100%). During the four-week follow-up, 17 complications with at least a possible link to the preceding LP procedure with the bioimpedance needle system were reported. The most common complications were various aches (head, back, stomach, limb) that were reported after seven procedures, vomiting/nausea after five procedures, PDPH after four procedures (linked to the same patient’s coincident LP procedures during the follow-up), and neutropenic infection after one procedure.

## Discussion

Traumatic lumbar puncture and leakage of blood into the spinal canal compromise the diagnostics of CNS involvement of acute leukemia and may have a negative impact on the patient survival. The present prospective study, conducted in the pediatric hemato-oncology departments of three university hospitals in Finland, demonstrated good clinical performance and safety of the novel bioimpedance system in a real-world setting. This pivotal study suggests clinical utility for the novel bioimpedance spinal needle system in the diagnostics and treatment of pediatric leukemia.

The study material comprised 152 intrathecal treatment procedures in 50 unselected pediatric patients with ALL who were recruited during their scheduled appointments to the hemato-oncology department; we omitted only the first diagnostic punctures because of ethical reasons. As around 50 Finnish children are annually diagnosed with ALL^26^, the patient material represents well the entire target population. Further, 26 pediatric oncologists, including both novice and highly experienced providers of LP, used the bioimpedance needle system, but only a few of them knew or had seen the device before the study. These physicians received a brief overview of the system and could try it with a lumbar phantom. Given the good results of the present study, the physicians seemed to adopt well the clinical use of the system with limited training. It is also notable that neither the physician’s experience in performing LPs nor the patient’s weight did essentially affect the success of the first attempt or incidence of TLP.

The success of LP at the first attempt achieved with the novel bioimpedance needle system was about 80%. Comparing this value to previous studies of pediatric patients with ALL is congruent with the 70% success rate achieved by seven experienced pediatricians with a conventional spinal needle^13^, the 90% success rate achieved by three pediatric oncologists with ultrasound guidance after specific training for its use^15^ and the 95% success rate without image guidance achieved by a single, highly experienced pediatric oncologist^14^. Clinically, the most relevant result of the present study is that the success of LP at the first attempt was significantly associated with almost four times lower incidence of TLP compared to LP procedures where two or more attempts were required. This finding not only underlines the importance of the first attempt in getting a high-quality CSF sample but also compares well to halved incidences of TLPs reported after successful first attempts in adults with leukemia or neurologic problems^16,27^, and children in general^17^. The present 10% incidence of TLP associated with the successful first attempts agrees well with the 16% incidence observed in a recent study where the overall success rate at the first attempt was 90%^15^. Sedation most likely accounts for the low incidence of TLP among the pediatric patients with ALL, but acceptable results may also be obtained in intrathecal treatment LPs without sedation^28^. In further considering the incidence of TLP, the present study did not include the diagnostic LPs, which may show a substantially lower incidence of TLP than later intrathecal treatment punctures (19% vs 32%)^2^, but not necessarily (20% vs. 18%)^9^. This issue warrants further studies.

Success at the first attempt is also likely to reduce the risk of PDPH^14^. In the present study, the incidence of PDPH was low, about 6%. Since the Quincke-type needle is associated with a higher incidence of PDPH compared to pencil-point spinal needles^29,30^, we compared the present incidence of PDPH only to studies of pediatric patients with ALL that were done with the 22G Quincke-type needle. Our finding is fully in line with the reported incidences of 7%^14^, 8%^11^, 11%^13^, and 14%^15^. The other post-procedural complications observed in this study were expected consequences of LP procedure or intrathecal treatment. Altogether, we found no indication that the use of the bioimpedance needle system would increase the patient risks.

The technical performance of the bioimpedance needle system was not perfect. Whereas the system can instantly detect the contact of the needle tip with CSF and provide the user with opportune audio-visual feedback to avoid too deep needle insertion, the user may unintentionally withdraw the needle slightly along with the stylet removal. If so, the initial contact between the needle lumen and CSF may vanish and explain the false CSF alarms, which took place in 9% of all study procedures. This value compares well with 11% and 19% incidences observed in previous clinical feasibility studies of the system^22,23^. Of the procedures completed with the system, the CSF detection sensitivity was 86%, being fully in line with the previously reported 82% in neonates and infants^23^ and 100% in adults^22^.

The bioimpedance needle system detects CSF when the continuously measured impedance stays sufficiently long time within the frequency-specific ranges of impedance classified as CSF. However, if some extrinsic (e.g., tissue remnants at the needle tip) or intrinsic factors (e.g., electrical contact) of the system disturbs the impedance measurement of the needle, the detection of CSF may be missed. According to the post-hoc analysis of recorded raw bioimpedance data and users’ comments on CRFs, about half of the missed CSF detections were attributable to soft tissue remnants that likely masked the needle tip electrode and altered the impedance values. The rest of the missed CSF detections were mostly due to deficiencies in the electrical contact. Despite the missed CSF detections, the success rate at the first attempt was high, and the LP procedure per se was not compromised. This finding pinpoints the intended purpose of the bioimpedance needle system as a guidance method providing supplemental information on the needle tip location while the final decision remains up to the physician.

Besides the bioimpedance needle system, another simple guidance method not requiring simultaneous imaging (e.g., ultrasound) is the early stylet removal from the spinal needle before penetrating the dura. In previous observational studies, the use of this technique has been associated with a higher success rate in pediatric patients^31,32^. However, there is not yet strong evidence that the early stylet removal technique would improve the success rate of LP but is under rigorous investigation^33^. Nevertheless, this technique seems to be frequently used at present: e.g., in a recent survey^34^, graduating residents utilized early stylet removal in more than 60% of their LP procedures in infants. This observation implies the novice physicians’ desire for objective information on the needle tip location when performing the LP procedure. The early stylet removal technique offers a direct way to see when CSF starts flowing through the needle whereas the bioimpedance needle system detects in real-time when the needle tip reaches contact with CSF without the stylet removal.

Single-arm design and observational nature are the main limitations of our study. Therefore, we cannot conclude that the novel bioimpedance needle system improved the success rate, reduced the incidences of TLP, PDPH or other complications, or was of added clinical benefit compared to the conventional spinal needle in the intrathecal treatment LPs of children with ALL. A randomized controlled trial would be the appropriate design to demonstrate the anticipated benefits. However, before conducting a demanding clinical trial with the system, we considered it essential to get first relevant information on the clinical performance and safety of the system in a sufficiently large and representative target group. Also, the prospective design is a strength in increasing the reliability of data collection and related findings.

## Conclusions

Pediatric oncologists adopted well the use of the novel bioimpedance spinal needle system in a real-world clinical setting. The system achieved a high success rate at the first attempt and a low incidence of TLP in a representative sample of pediatric patients with ALL. Also, the incidence of PDPH was particularly low, while the other complications were in line with the results reported in the literature. All these promising performance and safety results indicate clinical utility for the system in pediatric hemato-oncology. These results need to be confirmed in adequately powered randomized clinical trials where the bioimpedance spinal needle system is compared to a conventional spinal needle or other relevant comparators.

## Data Availability

The data that support the conclusions of this study are available from Dr. H. Sievanen, upon reasonable request considering the pertinent privacy and ethical restrictions.
